# Differences in health care utilisation between elderly from ethnic minorities and ethnic Dutch elderly

**DOI:** 10.1186/s12939-014-0125-z

**Published:** 2014-12-20

**Authors:** Ilona Verhagen, Wynand JG Ros, Bas Steunenberg, Wijnand Laan, Niek J de Wit

**Affiliations:** Julius Center for Health Sciences and Primary Care, University Medical Center Utrecht, Mailbox 85500, 3508 Utrecht, GA the Netherlands

**Keywords:** Health care usage, Ethnic minorities, Elderly

## Abstract

**Introduction:**

In the Netherlands, as in other Western countries, ethnic minority elderly are more often in poorer health than the indigenous population. The expectation is that this health disadvantage results in more frequent use of health care services.

**Methods:**

We studied registered data on the proportion of health care receivers, frequency of use, and health care costs collected by a major Dutch health insurance company in 2010. Data from 10,316 Turkish, 14,490 Moroccan, 8,619 Surinamese, and 1,064 Moluccan adults aged 55 years and older were compared with data from a sample of 33,725 ethnic Dutch older adults.

**Results:**

Unadjusted and adjusted (for age and gender) analyses showed the following. Moluccans had lower usage levels for all types of health care services. Use of primary health care facilities was higher for Turks, Moroccans, and Surinamese compared with the ethnic Dutch, with the exception that physical therapy was less frequently used among the Turks and Moroccans. Use of hospital care was lower, except for the Surinamese, who had a similar level of usage to that of the ethnic Dutch.

**Conclusions:**

The health disadvantage previously observed within most ethnic minority elderly populations does not result in an overall more frequent use of health care services. Further research is needed for the interpretation of the ethnic variations in health care use as potentially inequitable, by taking medical need, patient treatment preferences, and treatment adherence into account.

## Introduction

In the Netherlands, as in other Western countries, ethnic differences in health status exist among elderly [1]. Ethnic minority elderly are frequently disadvantaged: they are often in poorer health than the indigenous population This applies to self-reported health [2-5], mental health [6] as well as the prevalence of chronic diseases such as diabetes mellitus, COPD, musculoskeletal disorders, hypertension, and cardiovascular disease [3,7-9]. The incidence of many cancers is, however, lower. Although mortality is substantially lower, morbidity seems to be higher, in particular among those from the first-generation [10]. The expectation is that the health disadvantage observed within most ethnic minority elderly populations results in more frequent use of health care services. Therefore, we studied the health care utilisation of elderly from four large ethnic minority groups that have been in the Netherlands for decades: Surinamese, Turks, Moroccans, and Moluccans.

Despite their number is rapidly rising in the Netherlands recent research on this domain remains scarce. This is in line with the lack of international research on health care use within elderly ethnic minority populations as be observed in a recently published review [1]. Most recent Dutch studies are based on data collected in 2003 in a large sample of Surinamese, Turks, Moroccans, and Antillean aged 55 years and older [3,4]. These studies showed that GP service use to be higher whereas the use of outpatient hospital care, and physical therapy was lower to absent among the four ethnic minority elderly populations compared to ethnic Dutch elderly.

Clearly, there is a strong need for recent research on aspects of health care utilisation within ethnic minority elderly populations in the Netherlands. Instead of comparing health care utilisation by comparing the number of health care users or the frequency of health care use, as done in the previous studies, our study focusses on differences in health care utilisation by comparing health care costs. Additionally, we use data on the proportion of insured individuals receiving care and the frequency of use of health care provisions to gain insight into potential explanations for any differences in health care costs.

A difficulty with self-reported data on utilisation of health care is that there are indications that the cross-cultural validity of these data is suboptimal [11]. To study whether health care use of the four ethnic minority elderly populations in the Netherlands varies from the ethnic Dutch elderly, this study uses therefore data from registry data from the Achmea Health Insurance Company, the main health insurance company in the central part of the Netherlands.

## Methods

### The achmea health database

The Health Database of Achmea is based on reimbursement data for the provision of all medical care to 1,3 million insured patients in the central area of the Netherlands [12]. The database includes three domains of information: patients, health care professionals, and health care services provided. These three domains are linked to the patient’s individual identity. The database includes data on most of the health services provided by general practitioners (GPs) (e.g., type and date of GP consultations) and by other primary care professionals (e.g., chronic disease management programmes coordinated by practice nurses in primary care and physical therapy). In addition, it contains information on all drugs provided by pharmacists, such as the drug type (anatomical therapeutic codes (ATC codes)), dosage (defined and daily doses prescribed), prescriber, date of delivery, and cost. Medications provided by hospital pharmacies and over-the-counter medications are not registered in the database. The database also includes information on hospital services, such as hospital admissions, and diagnostic and therapeutic services per specialist provided in hospitals. For specialist services the diagnosis of each patient is also registered. The database contains information on all medical aids prescribed to treat a disorder or to help with a limitation (e.g., eyeglasses and hearing aids), except for medical aids provided while admitted and medical specialist treatment related to admission or treatment. The registration of pharmacy and other health care services provided has a high reliability and accuracy because health services are only reimbursed to the provider after the electronic data entry is extensively checked. Random checks and a comprehensive programme of material auditing are used to exclude coding errors [12]. Data are provided under the condition of anonymity. Each data request from researchers is assessed by a scientific committee on clinical and scientific relevance.

### Selection of subjects

All adults aged 55 years and older of Turkish, Moroccan, Surinamese (South-Asian Hindustan), and Moluccan origin were identified from the Achmea Health Database. The Dutch group was matched, according to age and gender, with the overall group of ethnic minorities.

Following methodology reported earlier, subjects from ethnic minorities were identified by their foreign nationality or last name [13,14]. First-generation Moroccans, Turks, and Surinamese were identified by their nationality because ethnicity is not included in the Achmea Health Database. Second-generation Moroccans, Turks, and Surinamese were identified by matching the last names of the selected first-generation adults with the remaining names in the database, as well as by visually identifying the origins of names [14]. The identification of first- and second-generation Moluccans was also based on their last names. Last names from the passenger list of all Moluccans who transferred to the Netherlands in 1951 (collected by the Moluccan Historical Museum in the Netherlands) were matched with the database and the origins of names were also visually identified.

### Characteristics of the groups studied

Together Surinamese, Turks, and Moroccans, accounted in 2010 for almost 2.5% of the total population of 55 years and older. Surinamese are the largest group of older non-Western ethnic minorities (51,321 in 2010), followed by Turks (35,014), Moroccans (32,489) [15]. Moluccans form the smallest group, but recent estimates including 2010 are not available. The number of older Moroccan men exceeds the number of Moroccan older women, while among the Surinamese the reverse is the case. The numbers of older Turkish men and women are about equal. The majority of older immigrants live in the large Dutch cities except for the Moluccans.

Older Turks and Moroccans generally have a low income. They are also poorly educated, and many older Turks and Moroccans (particularly women) have a poor command of the Dutch language.

The financial position of older Surinamese is generally slightly better than that of elderly Turks and Moroccans. Surinamese elderly also have a higher education level and generally have no problems with the Dutch language. Moluccans are the immigrant population with the lowest incidence of low income. The education level of this group is relatively high [16].

### Data extraction process

We analysed data from January 1, 2010, to December 31, 2010, which were the most recent and complete data available at the time of our data request. The variables selected from the Achmea Health Database included the following: all drugs dispensed by pharmacists (drugs dispensed by hospital pharmacies were not included), health services provided by GPs and other primary care professionals (such as physical therapists), and chronic disease management programmes coordinated by practice nurses in primary care. Other variables selected were hospital services (consisting of hospital admissions as well as diagnostic and therapeutic services) and medical aids provided for the treatment of medical conditions.

Socio-demographic variables included age (on January 1, 2010), gender, neighbourhood status (deprived or non-deprived), and type of health insurance. Deprived neighbourhoods were defined by postal codes [17]. We used the postal codes for deprived neighbourhoods as registered by The Dutch Healthcare Authority (NZa). Everyone who is registered as a resident of the Netherlands is obligated to have basic health insurance. In addition to the basic insurance, it is possible to purchase additional health insurance to cover services such as physical therapy and dental care. The type of health insurance was defined according to whether the insured person had basic health insurance or additional health insurance on January 1, 2010.

### Data analysis

We compared data from the four ethnic minority groups with those of a randomly sampled equally sized Dutch reference group with respect to mean costs per insured person for GP services, receipt of prescriptions, physical therapy, hospital services, and medical aids. Cost estimates were based on tariffs. Additionally, we investigated differences in the frequency of use and in the proportion of insured persons receiving these services.

Given the need to compare the ethnic groups in terms of arithmetic mean costs which is the most informative measure for cost data and statistical analysis based on transferring cost data or comparing medians using standard non-parametric methods may provide misleading conclusions [18], we used analyses of variance (ANCOVA) of untransformed costs whereby we adjusted for age and gender. ANCOVA is a method known to be fairly robust to non-normality especially if the sample size is large [19]. We performed also ANCOVAs to evaluate differences in frequency of use whereby we again adjusted for age and gender. An ANCOVA yielding significant results was followed by post hoc multiple comparison testing using the Bonferroni test.

Chi-square tests were performed to evaluate differences in the proportion of care users. Given the need to determine whether the results were affected by age and gender, we additionally performed separate group analyses by age and gender.

The statistical significance level was set at 0.05. All analyses were performed in SPSS 20.0 for Windows.

## Results

Data from 10,316 Turkish, 14,490 Moroccan, 8,619 Surinamese, and 1,064 Moluccan adults aged 55 years and older were compared with a sample of 33,725 ethnic Dutch individuals.

As shown in Table 1, the mean age ranged from 63.5 to 64.5 years. The Moroccans had a relatively high proportion of males (57.3%). A large proportion of Moroccans (40.9%), Turks (37.0%), and Surinamese (25.4%) lived in deprived neighbourhoods.

**Table 1:** **Socio-demographic characteristics**

	**Moroccan N = 14,490**	**Turkish N = 10,316**	**Surinamese N = 8,619**	**Moluccan N = 1,064**	**Dutch N = 33,725**	***p-*** **value**
Age (mean, SD)	64.1 (7.1)^1^	64.0 (7.1)^1^	64.5 (8.8)	63.6 (8.7)^1^	64.4 (7.8)	<0.0001
Gender (male) (%)	57.3^2^	49.4^2^	44.1^2^	46.2^2^	51.2	<0.0001
Living in a deprived neighbourhood (%)	40.8^2^	37.0^2^	25.4^2^	7.8	8.0	<0.0001
Additional health insurance (%)	92.7^2^	92.9^2^	87.6^2^	82.2^2^	91.0	<0.0001

^1^Mean differs significantly from that of the ethnic Dutch population (*p* < 0.05).

^2^Proportion differs significantly from that of the ethnic Dutch population (*p* < 0.05).

As shown in Table 2 (unadjusted), higher proportions of Turks, Moroccans, and Surinamese and lower proportions of Moluccans used GP services and received medication compared with ethnic Dutch older adults. The total proportion of Moroccans (29.5%) and Moluccans (25.2%) who received physical therapy was lower than that of the ethnic Dutch (30.7%). The percentages of Surinamese (65.3%) and Turks (63.6%) who used hospital services were higher than that of ethnic Dutch (59.8%). The proportions of Surinamese, Turks, and Moroccans using medical aids were significantly higher compared with the ethnic Dutch (34.7%). The overall results remained unaffected after performing separate group analyses by age and gender.

**Table 2:** **The unadjusted proportions of insured individuals receiving different health care services (2010)**

	**Moroccan N = 14,490**	**Turkish N = 10,316**	**Surinamese N = 8,619**	**Moluccan N = 1,064**	**Dutch N = 33,725**	***p*** **-value**
GP services (%)^2^	88.4^1^	90.4^1^	87.3^1^	79.9^1^	82.7	<0.0001
Receipt of prescriptions (%)^3^	92.2^1^	94.1^1^	89.8^1^	80.2^1^	85.4	<0.0001
Physical therapy (%)^4^	29.5^1^	30.9	33.4^1^	25.2^1^	30.7	<0.0001
Hospital services (%)^5^	60.1	63.6^1^	65.3^1^	48.5^1^	59.8	<0.0001
Medical aids (%)^6^	36.8^1^	38.8^1^	42.8^1^	28.5^1^	34.7	<0.0001

^1^Proportion differs significantly from that of the ethnic Dutch population (*p* < 0.05).

^2^Health services provided by general practitioners (GPs) and practice nurses in primary care.

^3^All medications dispensed by pharmacies (drugs dispensed by hospital pharmacies are not included).

^4^Health services provided by physical therapists.

^5^Hospital admissions and diagnostic and therapeutic services provided in hospitals.

^6^Medical aids prescribed to treat a disorder or to help with a limitation (except medical aids provided while admitted or medical specialist treatment related to admission or treatment).

Table 3 (adjusted for age and gender) summarises the mean individual use of health care services (±SE) among older adults from ethnic minority groups. The mean number of GP services and receipt of prescriptions per user showed similar trends; Turks, Moroccans, and specifically the Surinamese had higher numbers per user compared with the ethnic Dutch. The Moroccans (16.3 ± 0.3) and Turks (15.4 ± 0.3) received significantly fewer physical therapy sessions, compared with the ethnic Dutch (18.9 ± 0.2). The mean use of hospital services among Moroccans, Turks, and Moluccans did not differ from that of the ethnic Dutch. In contrary to the other ethnic minority groups, the Surinamese had a significantly higher number (9.0 ± 0.2) of medical aids compared with the ethnic Dutch (7.8 ± 0.1).

**Table 3:** **The adjusted mean number of health care interventions per user (2010)** ^**1**^

	**Moroccan N = 12,812**	**Turkish N = 9,326**	**Surinamese N = 7,521**	**Moluccan N = 850**	**Dutch N = 27,900**	***p*** **-value**
GP services (mean, SE)^3^	6.8	6.8	7.4	5.8	6.3	<0.0001
	(0.1)^2^	(0.1)^2^	(0.1)^2^	(0.2)	(0.0)	
	**Moroccan N = 13,359**	**Turkish N = 9,710**	**Surinamese N = 7,744**	**Moluccan N = 853**	**Dutch N = 28,793**	***p*** **-value**
Receipt of prescriptions (mean, SE)^4^	34.3	36.7	41.1	28.3	30.1	<0.0001
	(0.6)^2^	(0.7)^2^	(0.7)^2^	(2.2)	(0.4)	
	**Moroccan N = 4,268**	**Turkish N = 3,189**	**Surinamese N = 2,878**	**Moluccan N = 268**	**Dutch N = 10,360**	***p*** **-value**
Physical therapy (mean, SE)^5^	16.3	15.4	18.7	16.1	18.9	<0.0001
	(0.3)^2^	(0.3)^2^	(0.4)	(1.2)	(0.2)	
	**Moroccan N = 8,704**	**Turkish N = 6,561**	**Surinamese N = 5,626**	**Moluccan N = 516**	**Dutch N = 20,177**	***p*** **-value**
Hospital services (mean, SE)^6^	3.1	3.1	3.4	2.8	3.1	<0.0001
	(0.0)	(0.1)	(0.1)^2^	(0.2)	(0.0)	
	**Moroccan N = 5,331**	**Turkish N = 4,006**	**Surinamese N = 3,689**	**Moluccan N = 303**	**Dutch N = 11,704**	***p*** **-value**
Medical aids (mean, SE)^7^	7.5	6.9	9.0	6.8	7.8	<0.0001
	(0.2)	(0.2)^2^	(0.2)^2^	(0.7)	(0.1)	

^1^Adjusted for age and gender.

^2^Mean differs significantly from that of the ethnic Dutch population (*p* < 0.05).

^3^Health services provided by general practitioners (GPs) and practice nurses in primary care.

^4^All drugs dispensed by pharmacies (drugs dispensed by hospital pharmacies are not included).

^5^Health services provided by physical therapists.

^6^Hospital admissions and diagnostic and therapeutic services provided in hospitals.

^7^Medical aids prescribed to treat a disorder or to help with a limitation (except medical aids provided while admitted or medical specialist treatment related to admission or treatment).

As shown in Table 4 (adjusted for age and gender), the mean total costs per person (±SE) were lower for all ethnic minority groups, except the Surinamese (€3843.4 ± 87.2), compared with the costs for ethnic Dutch (€3473.3 ± 44.0). The mean costs per person for GP services and prescription medications showed similar trends; Surinamese, Turks, and Moroccans had higher costs than the ethnic Dutch. The mean costs per person for hospital services were lower for Turks (€1922.7 ± 70.5), Moroccans (€1854.3 ± 59.6), and Moluccans (€1472.1 ± 219.5) than for the ethnic Dutch (€2303.6 ± 39.0). The mean costs per person for physical therapy were lower for all minority groups, except the Surinamese, compared with the costs for ethnic Dutch. The Surinamese had higher mean costs per person for medical aids than the ethnic Dutch, but were lower for Turks, Moroccans, and Moluccans.

**Table 4:** **The adjusted mean costs of different health care services per insured persons (2010)** ^**1, 2, 3**^

	**Moroccan N = 14,490**	**Turkish N = 10,316**	**Surinamese N = 8,619**	**Moluccan N = 1,064**	**Dutch N = 33,725**	***p*** **-value**
Total costs (mean, SE)	3049.8 (67.3)^4^	3149.4 (79.6)^4^	3843.4 (87.2)^4^	2300.0 (247.9)^4^	3473.3 (44.0)	<0.0001
GP services (mean, SE)^5^	56.8 (0.5)^4^	58.7 (0.6)^4^	60.8 (0.6)^4^	42.0 (1.8)^4^	48.7 (0.3)	<0.0001
Receipt of prescriptions (mean, SE)^6^	819.4 (15.8)^4^	855.8 (18.7)^4^	871.2 (20.5)^4^	545.9 (58.3)^4^	727.2 (10.4)	<0.0001
Physical therapy (mean, SE)^7^	141.3 (3.4)^4^	139.3 (4.1)^4^	186.4 (4.5)	122.5 (12.7)^4^	176.7 (2.3)	<0.0001
Hospital services (mean, SE)^8^	1854.3 (59.6)^4^	1922.7 (70.5)^4^	2478.0 (77.2)	1472.1 (219.5)^4^	2303.6 (39.0)	<0.0001
Medical aids (mean, SE)^9^	178.1 (6.5)^4^	172.9 (7.7)^4^	247.0 (8.4)^4^	117.4 (24.0)^4^	217.2 (4.3)	<0.0001

^1^Adjusted for age and gender.

^2^The costs are indicated in Euros.

^3^Financial reimbursements of health care deliverables to care providers in the country of origin are included.

^4^Mean differs significantly from that of the ethnic Dutch population (*p* < 0.05).

^5^Mean costs of the health services provided by general practitioners (GPs) and practice nurses in primary care.

^6^Mean costs of all drugs dispensed by pharmacies (drugs dispensed by hospital pharmacies are not included).

^7^Mean costs of the health services provided by physical therapists.

^8^Mean costs of hospital admissions and diagnostic and therapeutic services provided in hospitals.

^9^Mean costs of medical aids prescribed to treat a disorder or to help with a limitation (except for medical aids provided while admitted or medical specialist treatment related to admission or treatment).

## Discussion

The health disadvantage previously observed within most ethnic minority elderly populations does not result in an overall more frequent use of health care services. Although we found that the use of primary health care facilities (GP services and medication prescriptions) within most ethnic minority groups is higher than for the ethnic Dutch, they generally make less use of hospital care, medical aids, and physical therapy. Our study confirms that older adults from ethnic minorities and ethnic Dutch older adults differ in health care utilisation. However, the pattern of differences is rather complex as the differences depend on ethnic group and type of health care.

The use of physical therapy was lower for the Moroccans and Turks. This finding was remarkably as older adults from these minorities are known to have a higher prevalence of musculoskeletal disorders [3]. The lower costs could be explained by the lower usage frequency of physical therapy. Despite the quite comparable number of therapy users among Moroccans and Turks compared with the ethnic Dutch, it seems that physical therapy care is discontinued sooner. This cannot be explained by reimbursement restrictions, such as not having the required supplemental insurance package, as most Moroccans and Turks did have additional health insurance (92.7% and 92.9%, respectively). It is possible that compliance with physical therapy is not optimal for the Turks and Moroccans due to unfamiliarity with this type of care and cultural discrepancies with regard to expectations and treatment preferences [20].

The costs of drugs dispensed by pharmacists were higher for the Moroccans, Turks, and Surinamese, which could be explained by a higher proportion of individuals in these groups receiving medication and by a greater number of prescriptions received per user. This higher number of dispensed prescriptions may be explained by the fact that patients from ethnic minorities more frequently expect a prescription in medical consultations [21] and have higher expectations from the effectiveness of pharmacotherapy. Another explanation may be the higher prevalence of chronic conditions and associated comorbidities that have been reported among these ethnic groups [3].

The costs of GP services were higher for the Moroccans, Turks, and Surinamese, which could be explained by the higher proportion of individuals in these groups who received GP care and by the slightly higher number of GP services they received. Higher use of primary care services may be related to an increased consultation rate due to poorer health perception, increased symptom alertness, and a higher prevalence of chronic disorders. Patients from ethnic minorities groups are more likely to consult their GP for minor issues [22], which may be due to insufficient knowledge of diseases and possibilities for self-care [23]. A third possible explanation is that language difficulties and cultural discrepancies with regard to expectations affect the quality of care and result in repeat consultations [24,25]. This explanation may be less applicable to the Surinamese, as they are generally fluent in the Dutch language.

Hospital care usage among Turkish and Moroccan older adults was lower than expected. The higher use of GP services combined with the relatively lower use of hospital care might be an indication that these ethnic groups are more inclined to consult their GP for minor complaints for which referral to hospital care is not required. It may also be that GPs refer patients from these groups to hospital care less often. A poorly experienced GP consultation due to language difficulties and cultural discrepancies may complicate the referral process.

The systematically lower health care usage among Moluccans compared with the ethnic Dutch probably reflects the better health status of this group, as has been reported in earlier studies [3].

Differences in cultural characteristics may have affected health care usage within these groups through health-related attitudes and beliefs that were learned during the process of acculturation. The way in which health problems are perceived are culturally determined and may have influenced decisions regarding health care use. Remarkably, not only did the Turks and Moroccans have a different pattern of health care usage than the ethnic Dutch, but so did the Surinamese and Moluccans. The latter groups are known to have a higher acculturation level and more self-identification with Dutch society than Turks and Moroccans. This may indicate that better acculturation does not automatically lead to a comparable health care use.

### Comparison with previous research

Although the number of studies conducted within ethnic minority elderly groups are limited, our findings are largely comparable regarding GP care, physical therapy, and hospital care [3,4,26]. The comparison of differences in medication use is complicated because we analysed the dispensed medications by pharmacists, while previous studies studied the actual utilisation as reported by the patient. Receipt of prescriptions does not necessarily lead to consumption of the medication and differences could well be a reflection of poor compliance with prescribed medication [27]. Nevertheless, we do not have indications that the differences in methods of data collection influenced our results. The number of outpatient prescriptions we found are comparable to self-reported use in previous studies [3].

### Limitations

There are several limitations to our study that need to be considered when interpreting our results.

A first limitation is that we could not assess whether the differences in health care utilisation as observed in our study can be interpreted as ethnic inequities in health care, either in accessibility or quality. We lacked information on medical need (the need for medical care based on a professional judgment about people’s health status) for health care, patient treatment preferences (ethnic groups may vary in their preferences for certain treatments), and treatment adherence, which is required for the interpretation of ethnic variations in health care use as potential inequitable [28]. Additionally, the consequences of the observed differences in health care use for health outcomes could not be analysed. Additional analysis of the consequences of the ethnic differences in health care use found for health outcomes might have provided insight into the potential inequity of these differences in health care use [28].

A second limitation was that cost estimates were based on tariffs instead of real costs. The actual costs could, therefore, have been lower of higher than the health care tariffs used in our study. Nevertheless, we did not have indications that a tariff for any of the health services did not represent an approximation that reflects the actual costs.

A third limitation was the ethnic Dutch group might include second-generation people with a Dutch father and a non-Dutch mother as a result of identification of ethnicity by last name (father’s last name). The same is true for Creole-Surinamese. They could not be identified by last name, because their last names are not sufficiently different from those of the ethnic Dutch. Given the large ethnic Dutch group included in our study, we assume that this has not affected our results.

A fourth limitation was the presence of outliers in our data. Patients with severe disease do have an increased use of health care services and induce above average costs. Because these outliers could have biased the results in our study, we recalculated the results after exclusion of the patients that were in the below 5% and above 95% range of total costs. This did not affect the results, the means and trimmed means showed similar patterns. Therefore, it is not likely that the outliers influenced our findings.

Finally, we did not provide information on over-the-counter medication, which could have influenced the results on the actual utilisation of medication. Such an influence, however, is not plausible because most medications in the Netherlands are dispensed based on prescriptions. Over-the-counter medications are limited to drugs such as aspirin, NSAIDs, H2 blockers, and laxatives [12].

## Conclusions

The health disadvantage previously observed within most ethnic minority elderly populations does not result in an overall more frequent use of health care services. Further research is needed for the interpretation of ethnic variations in health care use as observed in this study as potentially inequitable, by taking medical need, patient treatment preferences, and treatment adherence into account. We also recommend research on the consequences of the ethnic differences in health care use for health outcomes. This may provide insight into the presence of potential ethnic health care inequities.
